# Transparent and trustworthy CyberSecurity: an XAI-integrated big data framework for phishing attack detection

**DOI:** 10.3389/fdata.2025.1688091

**Published:** 2025-12-18

**Authors:** Muhammad Nauman, Hafiz Muhammad Usman Akhtar, Huseyn Gorbani, Muhammad Hadi Ul Hassan, Muhammad A. B. Fayyaz

**Affiliations:** 1 Faculty of Computing, The Islamia University of Bahawalpur, Bahawalpur, Punjab, Pakistan; 2 Queen Mary University of London, London, United Kingdom; 3 University of Bolton, Bolton, United Kingdom; 4 OTEHM, Manchester Metropolitan University, Manchester, United Kingdom

**Keywords:** CyberSecurity, cyber-attack detection, machine learning, explainable artificial intelligence, security paradigm

## Abstract

**Introduction:**

The exponential growth of heterogeneous, high-velocity CyberSecurity data generated by modern digital infrastructures presents both opportunities and challenges for threat detection, especially against increasingly sophisticated cyber-attacks. Traditional security tools struggle to process such data effectively, highlighting the need for scalable Big Data Analytics and advanced Machine Learning (ML) techniques. However, the black-box nature of many ML models limits interpretability, trust, and regulatory compliance in high-stakes environments.

**Methods:**

This study proposes an integrated framework that combines Big Data technologies, ML models, and Explainable Artificial Intelligence (XAI) to enable accurate, transparent, and real-time phishing attack detection. The framework leverages distributed computing and stream processing for efficient handling of large and diverse datasets while incorporating XAI methods to generate human-understandable model explanations.

**Results:**

Experimental evaluation conducted on four publicly available CyberSecurity datasets demonstrates improved phishing detection performance, enhanced interpretability of model decisions, and actionable insights into malicious URL behavior and patterns.

**Discussion:**

The proposed approach advances interpretable and scalable CyberSecurity analytics by addressing the gap between predictive accuracy and decision transparency. By integrating Big Data processing with XAI-driven ML, the framework offers a trustworthy solution for real-time threat detection, supporting informed decision-making and regulatory compliance.

## 1 Introduction

Advances in Information and Communication Technologies (ICT) and their pervasive integration into daily life have generated vast, rapidly evolving streams of data from highly diverse and heterogeneous sources. This exponential growth, driven by the proliferation of digital technologies and interconnected systems, creates unprecedented opportunities for innovation while simultaneously heightening the scope and complexity of CyberSecurity challenges. Modern digital infrastructures produce high-volume, high-velocity, and high-variety data originating from firewalls, intrusion detection systems (IDS), antivirus software, network traffic monitors, authentication records, and event logs—characteristics that strain the capabilities of traditional processing techniques (Balta et al., 2025; Neethirajan, 2025). CyberSecurity has consequently emerged as a core Big Data domain, with organizations generating terabytes of logs daily, often containing subtle indicators of malicious activity. Treating CyberSecurity data as “Big Data” enables the application of scalable analytics—such as distributed computing and Machine Learning (ML) to uncover hidden threats, generate actionable intelligence, and improve situational awareness (Afolabi et al., 2025; Sarker et al., 2020).

Big Data Analytics has become indispensable in modern CyberSecurity by facilitating the real-time processing and analysis of vast, heterogeneous datasets generated by digital infrastructures, including network traffic logs, system events, and external threat intelligence (Koca and Çiftçi, 2025; Kumar and Kundu, 2024). Traditional security tools are often inadequate for managing the scale, velocity, and complexity of such data, whereas Big Data technologies offer scalable storage solutions and advanced analytical capabilities for detecting anomalies, forecasting potential attacks, and identifying complex threat patterns. Analytical approaches such as ML, statistical modeling, and graph-based techniques enable security analysts to gain deep insights into attack vectors, insider threats, and zero-day vulnerabilities. The integration of these methods into Security Information and Event Management (SIEM) platforms enhances both the accuracy and efficiency of automated threat detection and incident response (Zuech et al., 2015; Iqbal et al., 2020).

The increasing complexity and interconnectivity of digital ecosystems have amplified both the sophistication and frequency of cyber threats (Zhang et al., 2022; Charmet et al., 2022). Concurrently, CyberSecurity systems generate massive, diverse, and fast-moving datasets that demand advanced analytical techniques for effective decision-making and timely countermeasure development. Artificial intelligence (AI) and ML are increasingly employed for anomaly detection, threat prediction, and real-time response. However, the opaque, “black box” nature of many ML models poses significant challenges for interpretability, accountability, and trust—issues that are particularly critical in high-stakes CyberSecurity contexts (Pawlicki et al., 2024). This has driven the adoption of XAI, which seeks to enhance the transparency of AI decision-making. By integrating XAI into CyberSecurity frameworks, stakeholders can better understand model reasoning, increase confidence in automated threat detection, and ensure compliance with ethical and regulatory standards (Mohale and Obagbuwa, 2025).

When applied together, ML and XAI enable advanced capabilities in cyber threat detection, malware classification, and fraud prevention (Shaukat et al., 2020). ML algorithms, including deep neural networks and anomaly detection models, can learn intricate patterns from large-scale CyberSecurity datasets, facilitating the identification of phishing attacks, malware variants, and zero-day exploits (Ferrag et al., 2020). XAI complements these capabilities by providing interpretable outputs that allow analysts to trace the rationale behind alerts, assess the reliability of predictions, and iteratively refine models. This interpretability is vital for compliance in regulated industries and for ensuring operational trust in mission-critical environments (Arrieta et al., 2020; de Bruijn et al., 2022). Consequently, XAI strengthens user confidence, enhances accountability, and improves operational adoption in sensitive sectors such as finance, healthcare, and law enforcement.

Despite the high detection accuracy of ML models, their black box characteristics hinder their broader deployment in critical infrastructure environments (Khan I. A. et al., 2024; Karim et al., 2023; Nauman et al., 2021a,b; da Silveira et al., 2025). To address this limitation, the present research proposes a combined approach that integrates Big Data Analytics, ML, and XAI to enhance phishing attack detection. The methodology leverages distributed computing and real-time stream processing to handle large-scale, heterogeneous datasets efficiently, while XAI techniques are employed to interpret model outputs in a human-understandable manner. The proposed framework is validated using four publicly available datasets, with performance evaluation focusing not only on detection accuracy but also on interpretability. By elucidating the decision logic of predictive models, the approach empowers cybersecurity professionals to identify structural patterns in malicious phishing URLs and to improve both detection capabilities and model training processes.

The novelty of this research lies in its integrated application of Big Data technologies, ML, and XAI for interpretable, scalable, and trustworthy cyber threat detection. Its main contributions are as follows:

1. Interpretation of black box ML models within a Big Data framework for cyber-attack detection.
2. Integration of XAI into ML based CyberSecurity systems to enhance transparency, interpretability, and trust in operational threat detection.
3. Utilization of cloud computing technologies for data storage and processing to enable pattern detection and pattern analysis from large, diverse datasets.
4. Combination of Big Data Analytics and XAI to produce human-understandable explanations for large-scale threat detection, empowering analysts to make transparent, evidence-based security decisions.

The article is organized as follows. Section 2 presents the Background. Then, Related work is discussed in Section 3. Afterwards, Section 4 discusses the Materials and Methods, Section 5 presents Discussions and finally, the Conclusion is presented in Section 6.

## 2 Background

### 2.1 Cyber-attacks

Intruders often launch cyber-attacks by creating fake websites and malicious links that mimic legitimate platforms to steal sensitive information, such as usernames, passwords, and banking details (SlashNext, 2022; IRONSCALES, 2025). These fraudulent URLs are typically delivered through deceptive emails, social media posts, or malicious advertisements. Attackers exploit weak password practices—like using default credentials or reusing passwords across multiple sites—to gain unauthorized access. Once users enter their data on these deceptive sites, attackers can hijack financial accounts, corporate systems, and personal information, often causing identity theft and financial loss (Agent, 2025). Compromised devices can join botnets used in Distributed Denial of Service attacks, flooding online services with excessive traffic and causing severe slowdowns or outages leading to financial impact and reputational damage (Securelist, 2023). To defend against these risks, experts recommend regular updates, strong and unique passwords, multi-factor authentication, and user awareness training to identify suspicious URLs and links.

Modern cyber-attacks are becoming more sophisticated, using AI-generated content to make fake websites and emails appear even more realistic (Amara and Salama, 2024). Attackers are now automating large-scale attacks using AI and ML, which makes traditional defense mechanisms less effective. This highlights the need for continuous investment in advanced threat detection systems, proactive monitoring, and regular CyberSecurity audits to stay ahead of emerging attack strategies.

### 2.2 Big data

The exponential rise in data generated by digital ecosystems including Internet of Things (IoT) devices, social media platforms, sensors, and enterprise systems has led to the emergence of Big Data as a transformative force in data-driven innovation. Big Data is typically characterized by the five V's: Volume, Velocity, Variety, Veracity, and Value, which collectively define its scale, complexity, and potential for actionable insights. Traditional data processing systems fail to cope with the magnitude and diversity of such datasets, necessitating the development of robust technologies such as Apache Hadoop (Nandimath et al., 2013), Apache Spark (Salloum et al., 2016), and NoSQL databases for efficient distributed storage and processing (Hashem et al., 2015; Hussain F. et al., 2023; Hameed et al., 2025b).

The strategic integration of Big Data technologies across various domains, including healthcare, finance, education, and CyberSecurity, has redefined decision-making frameworks by enabling predictive analytics, real-time monitoring, and automated intelligence extraction. In the healthcare sector, for example, Big Data analytics has significantly enhanced disease prediction, personalized treatment, and operational efficiency. A prominent study by Nauman et al. (2025) illustrates how Big Data is revolutionizing diabetes management by improving clinical decision-making and patient outcomes through intelligent data interpretation. As such, Big Data is no longer viewed merely as a technical or computational challenge, but rather as a strategic enabler of intelligent systems and evidence-based policies (Mughal and Hussain, 2025).

### 2.3 Explainable artificial intelligence

Explainable Artificial Intelligence XAI is a field dedicated to making the inner workings of ML models transparent and comprehensible to humans (Khan N. et al., 2024; Calzarossa and Massari, 2023; Arrieta et al., 2020; Gunning and Aha, 2019; Hameed et al., 2025a). As AI systems become increasingly complex and integrated into critical decision-making processes, the need for interpretability has become paramount (Khan I. A. et al., 2024). The XAI techniques aim to provide insights into how models make decisions, which is essential for validating their reliability and fairness. This transparency is crucial in high-stakes domains such as healthcare, finance, and autonomous systems, where opaque models can lead to untrustworthy outcomes and ethical concerns. Key methods in XAI include feature importance analysis, model distillation, and surrogate models, which collectively help demystify complex algorithms and build trust with end-users and stakeholders (Martins et al., 2024).

One prominent XAI technique is Local Interpretable Model-agnostic Explanations (LIME) (Hakkoum and Benjdira, 2020), which explains individual predictions of any classifier by approximating it locally with an interpretable model (Ribeiro et al., 2016). LIME generates perturbed samples around a given instance and learns a simpler, interpretable model to explain the predictions of the complex model. This approach has been widely adopted in various fields, including CyberSecurity, where it aids in detecting phishing attacks by highlighting the features that most influence the model's decision. Another technique, Shapley Additive exPlanations (SHAP) (Gianfagna and Di Nardo, 2021; Lundberg and Lee, 2017) leverages cooperative game theory to attribute the contribution of each feature to the model's output consistently and fairly (Albini et al., 2022). SHAP values provide a unified measure of feature importance, which helps compare and contrast the influence of different features across models. These XAI techniques enhance model transparency and empower users to understand, trust, and effectively manage AI systems.

## 3 Related work

### 3.1 ML in cybersecurity threat detection

The integration of AI and ML in CyberSecurity has significantly advanced threat detection, fraud prevention, and data protection. Recent studies have highlighted how AI-based solutions improve security in areas such as cloud computing, the IoT, and financial systems. For example, Hussain M. et al. (2023) developed a deep learning model for detecting ransomware that outperforms traditional signature-based methods. Similarly, Hoang et al. (2024) demonstrated how dimensionality reduction techniques enhance the performance of ML models in detecting Industrial IoT attacks at the network edge, reducing computational costs while maintaining accuracy. In addition, Imtiaz et al. (2025) proposed a deep learning framework for IoT intrusion detection based on optical networks, while Jouini et al. (2024) provided a detailed survey on ML frameworks that strengthen security in edge computing environments.

ML has become an essential part of CyberSecurity due to the increasing complexity and volume of modern cyber threats. Unlike traditional security methods, ML models can automatically detect unusual patterns and suspicious behaviors in real-time, helping to identify malware, unauthorized access, botnet activities, and other security breaches. These advanced models can analyze large datasets quickly, providing faster and more accurate threat detection compared to manual systems (Ali et al., 2023b; Usman et al., 2025a,b).

Recent studies have introduced various ML techniques to strengthen CyberSecurity systems. Researchers have successfully applied algorithms like XGBoost, CatBoost, and support vector machines to detect cyber threats in networks and IoT environments. These models achieved very high accuracy rates, often over 99%, on datasets like CIC-IDS2017 and CIC-IoT2023 (Usman et al., 2025c; Ahmad et al., 2024). The use of XAI has grown in popularity, helping security teams understand how these models make decisions, which improves trust and system reliability (Khan S. et al., 2024; Ali et al., 2023a).

A recent study also introduced a powerful detection system using a combination of autoencoders and XGBoost to effectively detect zero-day attacks, which are particularly difficult to identify (Usman et al., 2024). Overall, ML is now a critical tool in CyberSecurity, offering faster, smarter, and more adaptable protection against modern cyber-attacks.

ML also plays a significant role in detecting social engineering attacks, such as fraudulent websites and fake login portals that are designed to steal user credentials and sensitive information. By analyzing website structures, URL patterns, and user interaction data, ML models can effectively distinguish between legitimate and malicious websites. This proactive detection helps prevent users from falling victim to financial fraud, identity theft, and unauthorized account access (Khan S. et al., 2024).

In CyberSecurity for IoT systems, ML is particularly valuable due to the limited processing power and security measures in many connected devices. Modern approaches use lightweight ML models that can monitor traffic patterns, detect intrusions, and block suspicious activities without overwhelming the device's resources. This is essential for safeguarding smart homes, healthcare systems, and industrial environments where IoT devices are increasingly targeted by attackers (Usman et al., 2025a; Ahmad et al., 2024).

### 3.2 XAI in CyberSecurity

Explainable Artificial Intelligence (XAI) is becoming an important area of research in CyberSecurity. Researchers are focusing on XAI to improve the transparency and understanding of ML models used for CyberSecurity tasks (Tashtoush et al., 2024; Nwakanma et al., 2023; Zhang et al., 2022; Fan et al., 2024; Aslam et al., 2022). Traditional ML models are often complex and work like making it difficult for CyberSecurity experts to trust their decisions. XAI solves this problem by providing clear explanations of how models make predictions. This helps security professionals make better decisions and increases trust in automated CyberSecurity systems.

Tashtoush et al. (2024) developed a method to detect malicious URLs by using simple statistical features extracted from hexadecimal representations. This method, supported by ML and deep learning, also used XAI to select the most useful features, which reduced the training time and made the system more efficient. In another study, Fan et al. (2024) employed XAI to investigate how human behaviors and personal habits influence the likelihood of becoming a victim of CyberSecurity threats. Their model provided useful recommendations based on individual risk profiles and achieved good performance with 78% accuracy.

Aslam et al. (2022) introduced an XAI-based system to detect malicious domains using a large dataset. They found that the Extreme XGBoost model gave the best results, with an accuracy of 98.56%. By using XAI methods like SHAP and LIME, they were able to explain how the model made its decisions, thereby making the system more reliable and easier to understand. This approach helps CyberSecurity teams better analyze and improve their detection strategies.

XAI to CyberSecurity not only improves threat detection but also helps experts understand which features are most important in identifying malicious activity. Tools like LIME and SHAP show which patterns or data points lead to security alerts, making CyberSecurity systems more transparent and trustworthy. XAI also helps organizations meet legal and regulatory requirements by providing clear reasons for automated decisions. Overall, XAI is becoming an essential part of modern CyberSecurity solutions.

Recent studies show that Big Data plays a key role in improving the performance of CyberSecurity systems. Technologies like MapReduce and Apache Spark are commonly used to handle large datasets quickly and effectively. Combining Big Data Analytics with deep learning has led to better results in areas such as fraud detection, intrusion prevention, and system monitoring. This combination helps create intelligent, flexible CyberSecurity systems that can adapt to changing threats.

Table 1 summarizes recent studies in ML and explainable AI for CyberSecurity threat detection. Existing works have focused on applying Deep Learning and traditional ML classifiers across diverse security domains such as IoT, industrial control systems, and network intrusion detection. While these studies achieved high detection accuracy, most lacked interpretability and scalability in real-time environments. The present study addresses these limitations by integrating XAI methods with ML-based detection to enhance transparency and trust in cyber threat analytics.

**Table 1:** Summary of related studies on ML, XAI, and CyberSecurity threat detection.

**References**	**Method**	**Description**
Imtiaz et al. (2025)	Deep learning (CNN, LSTM)	Proposed a deep learning model for detecting intrusions in optical-network-based IoT systems.
Jouini et al. (2024)	ML algorithms (survey)	Surveyed ML frameworks for enhancing security in edge computing environments.
Hussain M. et al. (2023)	Deep neural networks	Developed a deep learning model that outperformed traditional signature-based ransomware detection techniques.
Hoang et al. (2024)	PCA + ML classifiers	Applied dimensionality reduction techniques to boost detection performance in Industrial IoT environments.
Usman et al. (2024)	Autoencoder + XGBoost	Proposed a hybrid model to effectively detect zero-day cyber-attacks.
Usman et al. (2025c)	Least squares SVM	Achieved high accuracy using LS-SVM on standard CyberSecurity datasets such as CIC-IDS.
Usman et al. (2025a)	Lightweight XGBoost	Introduced a real-time detection system for IoT CyberSecurity using lightweight ML models.
Ahmad et al. (2024)	Decision trees, SVM	Detected intrusions in smart home environments using CIC-IoT2023 dataset.
Tashtoush et al. (2024)	SHAP, LIME, ML/DL	Used statistical features from hexadecimal URLs with XAI to reduce training time and enhance explainability.
Fan et al. (2024)	Risk profiling + XAI	Evaluated how personal behavior impacts CyberSecurity risk; provided user-specific recommendations.
Aslam et al. (2022)	Extreme XGBoost + SHAP, LIME	Developed a highly interpretable domain detection system achieving 98.56% accuracy.
Khan S. et al. (2024)	Hadoop, spark + SHAP	Combined Big Data platforms with explainable ML models for scalable intrusion and fraud detection.
Usman et al. (2025b)	Systematic review of ML	Reviewed recent ML approaches for CyberSecurity threat modeling and detection.

## 4 Materials and methods

**Figure 1:**
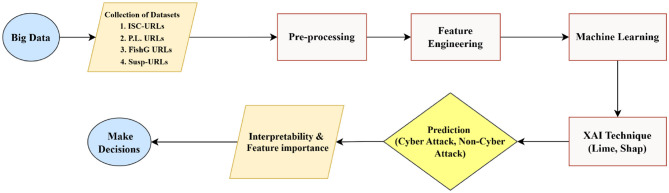
Flowchart of cyber-attack detection using black box ML and XAI.

The flowchart in Figure 1 illustrates the proposed approach for Cyber-Attack detection which integrates black box ML techniques with XAI techniques. The process begins with the collection of multiple publicly available URL datasets, including ISCX-URLs, PL-URLs, FishG URLs, and Suspicious URLs, covering both Cyber-Attack (Malicious) and Non-Cyber Attack (Benign) categories. The data undergoes pre-processing to remove inconsistencies and extract meaningful patterns, followed by feature engineering to transform raw URLs into structured feature sets. The ML models are then trained to classify URLs as Cyber-Attack or Non-Cyber Attack. Post-classification, XAI techniques such as LIME and SHAP are applied to interpret model predictions, highlighting the most influential features contributing to each decision. Finally, the interpretability results help to make informed decisions by providing transparency into the internal decision-making process of the model.

**Figure 2:**
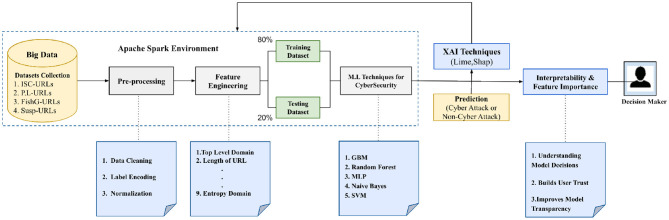
The proposed approach for cyber-attack detection using ML and XAI integration in big data environment.

The proposed method, illustrated in Figure 2, is applied to detect cyber-attacks and explain crucial features in URLS for decision-making processes. This study elucidated the significance of features for internal decision-making processes by applying LIME. The presented approach encompasses three significant steps: first, feature engineering from raw URLs; second, training ML models in Big Data environment; and third, employing LIME techniques to interpret the models and visualize their internal decision-making processes. All ML models and their corresponding datasets are publicly accessible via the following link.^1^

### 4.1 Data collection and pre-processing

To demonstrate the effectiveness of the proposed approach was applied to four publicly available datasets. Table 2 describes the datasets used in this study for Cyber-Attack detection, including their year of release and the number of Cyber-Attack and Non Cyber-Attack URLs. The ISCX-URLs dataset, available at https://www.unb.ca/cic/datasets/url-2016.html, contains 9,964 Cyber-Attack and 35,372 Non Cyber-Attack URLs. It is a comprehensive and diverse collection that includes spam, phishing, malware, and benign URLs, making it well-suited for both training and evaluation. The second dataset, PL-URLs, available at https://data.mendeley.com/datasets/j43jtv3zzc/1, consists of 698 Cyber-Attack and 205 Non Cyber-Attack URLs. The third dataset, PhishGuard URLs, accessible at https://data.mendeley.com/datasets/vfszbj9b36/1, includes 25,100 Cyber-Attack and 35,017 Non Cyber-Attack URLs. Its larger size and diverse content make it suitable for evaluating model generalization and robustness. The final dataset, Suspicious-URLs, published in Frontiers in Computer Science with DOI doi: 10.3389/fcomp.2024.1308634, consists of 25,000 Cyber-Attack and 25,070 Non Cyber-Attack URLs. Its balanced distribution offers a reliable benchmark for assessing model performance against modern phishing threats.

**Table 2:** Summary of datasets utilized in this study, including original and SMOTE-balanced versions.

**Sr#**	**Dataset**	**Year**	**Cyber-attack instances**		**Non-cyber attack instances**	
			**Original**	**SMOTE**	**Original**	**SMOTE**
1	ISCX-URLs	2016	9,964	35,322	35,372	35,372
2	P.L-URLs	2024	698	698	205	695
3	PhishGuard URLs	2024	25,100	34,876	35,017	35,008
4	Suspicious-URLs	2023	25,000	–	25,070	–

The datasets utilized in this study exhibited varying degrees of class imbalance, with Cyber-Attack URL instances being considerably fewer than Non Cyber-Attack URLs, particularly within the ISCX and PhishGuard datasets. To mitigate this imbalance and prevent the models from becoming biased toward the majority class, the Synthetic Minority Over-sampling Technique (SMOTE) was employed to generate synthetic samples of the minority class, resulting in a more balanced and representative training dataset. SMOTE is a data augmentation technique that synthesizes new minority class samples by interpolating between existing instances, thereby equalizing class distributions. This method was selected due to its proven effectiveness in addressing class imbalance, improving model generalization, and reducing bias toward the dominant class.

These datasets collectively provide a diverse and robust foundation for evaluating the proposed Cyber-Attack detection methodology, allowing for a comprehensive assessment of model performance across varying data distributions, URL types, and timeframes. Effective data pre-processing is essential to ensure reliable results; insufficient preparation can introduce biases and inconsistencies that may lead to inaccurate conclusions. The pre-processing phase involves several key steps, including data cleaning, integration, reduction, and discretization. In the context of phishing detection, the primary goal of pre-processing is to reduce false positives and false negatives, thereby enhancing model accuracy and reliability.

During pre-processing, data cleaning and feature scaling were applied to enhance data quality and ensure model effectiveness. Feature scaling normalizes all input features to a common range, preventing any single attribute from dominating the learning process due to scale differences. This standardization promotes balanced model training, improves convergence during optimization, and contributes to more consistent and accurate performance across datasets.

After pre-processing, all datasets were merged, and stratified splitting was applied to preserve the class distribution of Cyber-Attack and Non Cyber-Attack URLs. The final data division followed an 80%/20% ratio, with 80% used for model training and 20% reserved exclusively for testing. Random seeds were fixed for all splits to ensure full reproducibility. This partitioning strategy provides balanced evaluation and prevents bias introduced by uneven sampling or temporal overlap.

Effective data pre-processing is essential to ensure reliable results; insufficient preparation can introduce biases and inconsistencies that may lead to inaccurate conclusions. The pre-processing phase, therefore, encompasses cleaning, integration, reduction, discretization, and normalization, collectively enhancing model stability, improving convergence, and reducing false positives and false negatives in phishing URL classification.

### 4.2 Experimental setup

In an experimental study, we utilized Google Colab (Bisong, 2019) and Google Drive (Quick and Choo, 2014) as our cloud computing platforms to perform phishing and cyber-attack detection experiments, leveraging Python for scripting and Apache Spark for processing large-scale URL datasets. Specifically, the Big Data Analytics framework was implemented using Python (Van Rossum et al., 1995) on Google Colaboratory. PySpark 3.3.0 was employed to build and manage the data pipeline, demonstrating the seamless integration of cloud resources and Big Data tools in CyberSecurity driven projects.

### 4.3 Feature engineering

Feature engineering is an important step in the ML process. It means creating or changing new features to help the model perform better. The goal is to turn raw data into a form the model can understand more easily, which helps it make more accurate predictions. This can include scaling numbers, converting categories into numbers, combining features, or adding new features based on expert knowledge. In short, feature engineering helps give the model the best possible information to learn from.

### 4.4 Feature engineering algorithms

In this research, to better analyze the structure and behavior of URLs, nine features were extracted from the datasets. These features focus on the basic structure, such as the number of dots, delimiters, and URL length, and more advanced indicators, like domain entropy and sensitive words. Table 3 provides an overview of these features and brief descriptions, highlighting how each can help identify potentially malicious URLs.

**Table 3:** Overview of engineered features from the datasets along with their descriptions.

**Sr**.	**Feature**	**Description**
1	Top-level domains	Counts the number of TLDs in the URL, which can reveal compound or redirected domains.
2	Length of URL	Measures the total character count of the URL, which may suggest its complexity or intent.
3	Digits in query	Counts digits in the URL's query string, potentially indicating tracking or complex data manipulation.
4	No. of dots in URL	Counts periods in the URL, which can reflect the structure and potential for obfuscation.
5	No. of delimiters in domain	Counts delimiters in the domain, which can indicate attempts to manipulate the domain's appearance.
6	No. of delimiters in path	Counts delimiters in the URL path, suggesting the depth and complexity of the URL structure.
7	Length of longest token in path	Measures the longest segment in the URL path, which may house significant or suspicious elements.
8	Domain length	Measures the character count of the domain name, which can be indicative of the site's legitimacy.
9	Domain entropy	Assesses the randomness of the domain name, with higher entropy potentially indicating a malicious domain.

Feature engineering often involves breaking down raw data into useful components. For instance, in attack detection, a URL like http://abc.edu/last/ can be tokenized into parts such as [“http,” “abc,” “edu,” “last”]. These tokens help identify patterns that distinguish malicious URLs from legitimate ones. In the proposed approach, the first step was to break the URL into smaller parts. This process is called tokenization. Breaking the URL like this helped in finding useful patterns to check if a URL is safe or dangerous. These parts (tokens) are then used to make a feature vector for the ML model. This helps the model detect cyber-attacking websites better. For instance, in Algorithm 1, the tokenize function creates a list called tokenized_list that stores all the parts. The total number of parts is saved in D, and the feature vector keeps track of each part and its position.

Algorithm 1 Count the number of tokens in the URL.

 Require:  URL
 Ensure:  the Number of token counts in the URL
 1:  function tokenCount(URL)
 2:   *count*←0
 3:   *index*←0
 4:   *Tokenized*_*list*←*tokenize*(*URL*)
 5:   for all token in *Tokenized*_*list* **do**
 6:   *count*←*count*+1
 7:   *D*[*index*]←*count*
 8:   end **for**
 9:  end **function**

The computational procedures for the nine selected features are detailed in the following sections to provide a comprehensive overview of the feature engineering process.

#### 4.4.1 Top-level-domain

In recent observations, attackers have utilized multiple top-level domains (TLDs) within a single domain name to obfuscate their true intentions. This tool systematically tracks the number of TLDs present in a given URL. The extraction of this feature from URLs is outlined in Algorithm 2. In our methodology, we monitored a comprehensive list of 920 TLDs, denoted as *P* = [*P*_1_, *P*_2_, *P*_3_, …, *P*_*y*_].

Algorithm 2 Find number of top-level domains in URL.

 Require:  URL
 Ensure:  Number of top-level domains in URL
 1:  function find_domain(URL)
 2:   *P*←{*P*_1_, *P*_2_, ..., *P*_*n*_} ⊳ List of known TLDs
 3:   *count*←0
 4:   *index*←0
 5:   *d*← find_top_level_domain(URL)
 6:   for all domain in *d* **do**
 7:   if domain ∈*P* **then**
 8:   *count*←*count*+1
 9:   *D*[*index*]←*count*
 10:   end **if**
 11:   end **for**
 12:  end **function**

While a standard URL *u* is expected to contain a single TLD, represented as *d* = [*d*_1_, *d*_2_, *d*_3_, …, *d*_*n*_], there are cases where a URL may incorporate multiple TLDs. For each segment *d*_*k*_ in the domain, where *d*_*k*_ is any potential TLD, it is checked against the list *P*. The algorithm proceeds by extracting the TLD from the URL and determining the frequency of its occurrence. The valid TLDs identified at each step are stored in the list *D*. The presence and count of each TLD are then recorded in dataset *D*, providing a measure of TLD multiplicity in the analyzed URLs.

It is observed that attackers often use multiple top-level domains (TLDs) in a single URL to hide their intent. An algorithm extracts and counts TLDs from URLs by checking each segment against 920 valid TLDs. The identified TLDs are recorded, helping detect suspicious patterns in malicious URLs.

#### 4.4.2 Length of URL

Malicious URLs tend to be significantly longer than legitimate ones, as attackers often incorporate multiple domain names and extended links to obfuscate the URL's true nature and intentions. This deliberate manipulation increases the overall length of the URL, making it more challenging for users and security systems to detect malicious activity. Algorithm 3 calculates the total length of the given URL by systematically counting every character within it.

Algorithm 3 Calculate length of URL.

 Require:  URL
 Ensure:  Length of URL
 1:  function length(URL)
 2:   *length*←0
 3:   *index*←0
 4:   for all character in URL **do**
 5:   *length*←*length*+1
 6:   *D*[*index*]←*length*
 7:   end **for**
 8:  end **function**

The algorithm counts each character in the URL to determine its total length, helping to identify unusually long URLs that may indicate malicious activity.

#### 4.4.3 Digits in query

The Algorithm 4 tokenizes the text and appends a question mark before searching for numeric values within the query string of a URL. The notation *Q* = [*q*_1_, *q*_2_, *q*_3_, …, *q*_*n*_] indicates that the algorithm may encounter multiple queries associated with a single URL *u*. The set *X* = [*x*_1_, *x*_2_, *x*_3_, …, *x*_*n*_] represents the sum of all numeric values found within each query in *Q*.

Algorithm 4 Number of digits in query string.

 Require:  URL
 Ensure:  the Number of digits in a query string
 1:  function digits_in_query_string(URL)
 2:   *y*←0
 3:   *index*←0
 4:   *Q*← query_string(URL)
 5:   if *Q* is not empty **then**
 6:   for all query in *Q* **do**
 7:   *X*_*k*_← digits_in_query_string(query)
 8:   *y*←*y*+*X*_*k*_
 9:   end **for**
 10:   *D*[*index*]←*y*
 11:   else
 12:   *D*[*index*]←−1
 13:   end **if**
 14:  end **function**

The function responsible for counting digits within these queries calculates the total number of digits across all elements of *Q*. For a given URL *u*, let *Y* denote the total number of characters. The sum *x*_*k*_ for each query *k* is determined by summing the digits found in that specific query. This relationship can be mathematically defined as follows:


$$\begin{array}{l} Y=\overset{n}{\underset{k=1}{\sum }}, \end{array}$$ (1)

where n is an arbitrary number.

#### 4.4.4 Number of dots in URL

The number of dots in a URL can be an important indicator of its authenticity. Malicious websites are observed to often use more dots to create the illusion of legitimacy, deceiving users into believing that they are visiting a genuine site. For example, a legitimate URL like “http://www.abc.com” could be manipulated to appear as “http://www.ab.c.com,” misleading users. Recognizing and being cautious of URLs with unusual dot patterns is key to avoiding phishing scams.

#### 4.4.5 Number of delimiters in domain

Attackers sometimes employ special characters, such as “–” or “+,” to make a malicious URL appear legitimate. For example, while the legitimate URL might be “http://www.abc.com/,” a malicious variant could be crafted as “http://www.ab+c.com” to deceive users into thinking it is authentic. Recognizing these patterns and understanding the role of such delimiters is crucial in identifying and avoiding malicious attempts.

In this method, six characters—specifically “.”, “;”, “{”, “|”, and “+”—are used as separators to identify and parse different URL segments. The find-available-domain method determines the number of domains within a phishing URL by comparing these characters to the set *V*. The delimiter-count method increases the count *D* each time a match is found between the characters in the URL and the set *V*.

#### 4.4.6 Number of delimiters in path

When comparing URLs, it was observed that malicious URLs often contain twice as many spaces between words compared to legitimate URLs. Malicious URLs are observed to frequently include potentially harmful files, such as “.png” or “.js,” which follow delimiters, facilitating malicious activities. In the proposed Algorithm 6, the “find-available-path” method identifies the total number of path segments in a URL, while the marker count function tracks the locations where these breaks occur within the path. This analysis is critical for detecting the subtle differences in URL structure that indicate phishing attempts.

#### 4.4.7 Length of the longest token in path

Algorithm 5 outlines the procedure for identifying the longest path within a given URL. In general, Attackers often conceal malicious content within long and complex URL codes, making it significantly more time-consuming to access phishing URLs—approximately three times longer than accessing legitimate URLs. The find-paths function in Algorithm 6 identifies multiple paths within the URL list *G*, while the find-length function calculates the length of each path, storing the longest path length in *H*. For URLs that do not contain a valid path, a value of “-1” is recorded in dataset *D*. This approach helps detect URLs with suspiciously long and convoluted structures, often indicative of phishing attempts.

Algorithm 5 Length of the longest token in the path.

 Require:  URL
 Ensure:  Length of the longest token in the URL path
 1:  function longest_path(URL)
 2:   *index*←0
 3:   *G*← find_paths(URL)
 4:   if *G* is not empty **then**
 5:   Initialize empty list *F*
 6:   for all path in *G* **do**
 7:   *f*_*k*_← find_length(path)
 8:   Append *f*_*k*_ to *F*
 9:   end **for**
 10:   *H*←max(*F*)
 11:   *D*[*m*_7_][*index*]←*H*
 12:   else
 13:   *D*[*m*_7_][*index*]←−1
 14:   end **if**
 15:  end **function**

Algorithm 6 Number of delimiters in path and domain.

 Require:  URL
 Ensure:  The Number of delimiters in the path and the domain
 1:  function DelimiterCount(input)
 2:   *V*←{'@', '/', ' = '}
 3:   *count*←0
 4:   for all character in input **do**
 5:   if character ∈*V* **then**
 6:   *count*←*count*+1
 7:   end **if**
 8:   end **for**
 9:   return *count*
 10:  end **function**
 11:  function NumberOfDelimiter(URL)
 12:   *C*_1_←0, *C*_2_←0
 13:   *pathIndex*←0, *domainIndex*←0
 14:   *G*← findAvailablePath(URL)
 15:   *R*← findAvailableDomain(URL)
 16:   if *G* is not empty **then**
 17:   for all path in *G* **do**
 18:   *count*← DelimiterCount(path)
 19:   *C*_1_←*C*_1_+*count*
 20:   *D*[*m*_6_][*pathIndex*]←*C*_1_
 21:   end **for**
 22:   else
 23:   *D*[*m*_6_][*pathIndex*]←−1
 24:   end **if**
 25:   if *R* is not empty **then**
 26:   for all domain in *R* **do**
 27:   *count*← DelimiterCount(domain)
 28:   *C*_2_←*C*_2_+*count*
 29:   *D*[*m*_5_][*domainIndex*]←*C*_2_
 30:   end **for**
 31:   else
 32:   *D*[*m*_5_][*domainIndex*]←−1
 33:   end **if**
 34:  end **function**

#### 4.4.8 Domain length

Attackers often use elongated domains to create the appearance of a legitimate URL, while genuine URLs typically feature shorter, more concise domains. This feature compares the domain lengths of authentic and fake URLs. By determining the domain length of each URL and storing these values in a collection, we can analyze the differences between complete and fraudulent URLs. This comparison helps identify potentially deceptive domains artificially lengthened to mislead users.

For instance, attackers often extend domain names to disguise phishing URLs as legitimate. This method compares the length of domains in genuine and suspicious URLs, helping to identify fraudulent ones. The algorithm counts delimiters in the domain and path to detect unusual patterns.

#### 4.4.9 Domain of entropy

Domain Entropy quantifies the unpredictability or randomness of characters within a single domain string extracted from a URL. Unlike the previous version, which computed entropy across a list of domains, this revised approach calculates entropy based on the character frequency distribution of each domain. High entropy values indicate greater randomness—often associated with obfuscated or algorithmically generated phishing domains—while lower entropy values are typical of legitimate, human-readable domains. The entropy *H* is calculated using the standard information-theoretic formula:


$$\begin{array}{l} H=-\overset{n}{\underset{i=1}{\sum }}p_{i}\log_{2}\left(p_{i}\right) \end{array}$$ (2)

where *p*_*i*_ is the probability of occurrence of the *i*^*th*^ character in the domain string.

The updated computation process is illustrated in Algorithm 7.

Algorithm 7 Character-level domain entropy.

 Require:  URL
 Ensure:  Entropy value of the domain string
 1:  function Calculate_Entropy(URL)
 2:   *domain*← extract_domain(URL)
 3:   if *domain* is not empty **then**
 4:   Initialize empty dictionary *frequency*
 5:   for all character *c* in *domain* **do**
 6:   Increment *frequency*[*c*] by 1
 7:   end **for**
 8:   *entropy*←0
 9:   *total*_*chars*← length of *domain*
 10:   for all *freq* in *frequency*.*values*() **do**
 11:   *prob*←*freq*/*total*_*chars*
 12:   *entropy*←*entropy*−*prob*×log_2_(*prob*)
 13:   end **for**
 14:   return *entropy*
 15:   else
 16:   return 0
 17:   end **if**
 18:  end **function**

Entropy evaluates the randomness of domain names in a URL by analyzing their frequency distribution. For example, domains are extracted from the URL, their occurrences are counted, and probabilities are calculated. Based on these probabilities, the entropy is determined, with higher values indicating more unpredictability. This measure helps identify patterns that are useful for detecting malicious URLs.

Domain Length Density evaluates the average length of domains in a URL. Unusually long domains may indicate phishing. The algorithm sums the lengths of all domains and divides them by the number of domains to calculate the average. If no domains are found, it returns –1.

### 4.5 Hyper-parameter configuration

To ensure fairness and reproducibility, all models were trained and optimized under uniform experimental conditions. The dataset was divided using an 80:20 stratified split, maintaining the ratio of Cyber-Attack and Non Cyber-Attack URLs across all subsets. All experiments were executed on Google Colab connected with Google Drive, using Python 3.0 and PySpark 3.3.0 as the primary data processing and ML framework.

Hyperparameter tuning was performed using PySpark's CrossValidator with five-fold cross-validation and a defined parameter grid. For the Random Forest model, the grid included numTrees∈{10, 20, 30} and maxDepth∈{5, 10, 15}. The best-performing configuration was selected based on validation accuracy. This approach ensured a transparent, data-driven model selection process while preventing overfitting. All random seeds were fixed, and pre-processing and pipeline scripts were documented for reproducibility. Table 4 summarizes the final hyperparameter settings used in this study.

**Table 4:** Summary of optimized hyper-parameters for each learning algorithm.

**Sr**.	**Model**	**Key hyper-parameters and settings**
1	GBM	Learning rate (0.05); Number of estimators (300); Max depth (5); Subsample (0.8); Loss function (”deviance”).
2	Random Forest	Number of trees ([10, 20, 30]); Max depth ([5, 10, 15]); Criterion (”gini”); Evaluated using 5-fold cross-validation with PySpark's CrossValidator.
3	MLP	Hidden layers ([64, 32]); Activation (ReLU); Learning rate (0.001); Optimizer (Adam); Batch size (64); Epochs (100).
4	Naïve Bayes	Smoothing parameter (var_smoothing = 1e-9).
5	SVM	Kernel (RBF); Regularization (C = 1.0); Gamma (scale).
6	LIME explainer	Number of perturbations (5,000); Kernel width (0.75); Distance metric (Euclidean).
7	SHAP explainer	Background sample size (1,000); Explainer type (TreeExplainer); Maximum samples (5,000).

### 4.6 Performance metrics

The ML models were applied to categorize URL instances either classified as “Cyber-Attack” or “Non Cyber-Attack.” The “Cyber-Attack” label represents malicious URLs that pose potential threats to users, while “Non Cyber-Attack” refers to safe URLs suitable for browsing. To evaluate the performance of the classification models, a confusion matrix was computed, as shown in Table 5. A confusion matrix provides a summary of prediction results, consisting of four components: True Positives (TP), False Positives (FP), False Negatives (FN), and True Negatives (TN). TP represents correctly identified Cyber-Attack instances, FP refers to Non Cyber-Attack instances incorrectly classified as Cyber-Attack, FN occurs when Cyber-Attack are missed, and TN corresponds to correctly identified Non Cyber-Attack instances.

**Table 5:** Confusion matrix.

		**Predicted class**	
		**Cyber-attack**	**Non cyber-attack**
Actual class	Cyber-attack	TP	FN
	Non cyber-attack	FP	TN

Several key parameters were selected to evaluate and compare the classification model's precision. These include the number of TP, TN, FP, and FN. Metrics such as Accuracy (ACC), Precision (P), Recall (R), Sensitivity (TPR), Specificity (TNR), and the Area Under the ROC Curve (AUC) were used as designated quantitative measures. The total number of instances is denoted as *N*, and these metrics are computed accordingly to provide a comprehensive assessment of the model's performance. The value *N* can be calculated as:

The value *N* can be calculated as:


$$\begin{array}{l} \text{N = TP + TN + FP + FN} \end{array}$$ (3)

True positive rate (TPR) or sensitivity: also known a Recall (R), refers to the proportion of true positives correctly identified out of all actual positives. It indicates how well the model identifies positive cases.


$$\begin{array}{l} \text{TPR}=\frac{\text{TP}}{\text{TP}+\text{FN}} \end{array}$$ (4)

False positive rate (FPR): also known as the fall-out, measures the proportion of actual negatives that are incorrectly identified as positives and is defined as:


$$\begin{array}{l} \text{FPR}=\frac{\text{FP}}{\text{FP}+\text{TN}} \end{array}$$ (5)

False negative rate (FNR): also known as the miss rate, measures the proportion of actual positives that are incorrectly identified as negatives and is defined as:


$$\begin{array}{l} \text{FNR}=\frac{\text{FN}}{\text{FN}+\text{TP}} \end{array}$$ (6)

Precision (P): precision tells you how accurate the model's positive predictions are. It is the proportion of true positives among all positive predictions.


$$\begin{array}{l} \text{P}=\frac{\text{TP}}{\text{TP}+\text{FP}} \end{array}$$ (7)

Area under the ROC curve (AUC): the ROC curve graphically displays the predictive model's performance by plotting the actual positive rate against the false positive rate for various threshold settings. AUC represents the likelihood that the model will rank a randomly chosen positive instance higher than a randomly chosen negative one. The perfect prediction value for AUC is 1.0, and 0.5 corresponds to the diagonal on the ROC curve. F-measure (F1 score): the harmonic mean of Precision and Recall. It balances the two metrics, especially when there is an uneven class distribution.


$$\begin{array}{l} \text{F1 Score}=\frac{2\times \text{(P)}\times \text{(R)}}{\text{(P)}+\text{(R)}} \end{array}$$ (8)

Accuracy (ACC): the number of correct predictions a classification algorithm makes is called its accuracy. To find it, divide the number of correct predictions by the total number of data points.


$$\begin{array}{l} \text{Accuracy}=\frac{\text{(TP)}+\text{(TN)}}{\text{(TP)}+\text{(TN)}+\text{(FP)}+\text{(FN)}} \end{array}$$ (9)

These parts impact important metrics. Accuracy shows how often the model is right overall. Precision measures how many of the positive predictions are correct. Recall shows how many actual positive cases the model identifies. The F1-score balances precision and recall, especially with imbalanced data. Specificity measures how well the model avoids false alarms. These insights help improve the model's performance.

### 4.7 Results

The results presented in Tables 6, 7 highlight the comparative performance of the evaluated ML algorithms across four benchmark datasets ISCX-URLs, PL-URLs, PhishGuard URLs, and Suspicious-URLs. The proposed approach demonstrated strong and consistent results, with the Gradient Boosting Machine and Random Forest models outperforming other classifiers in most performance metrics.

**Table 6:** Performance comparison of TPR, TNR, FPR, and FNR across datasets used in the study.

**Model**	**ISCX-URLs**				**PL-URLs**				**PhishGuard URLs**				**Suspicious-URLs**			
	**TPR**	**TNR**	**FPR**	**FNR**	**TPR**	**TNR**	**FPR**	**FNR**	**TPR**	**TNR**	**FPR**	**FNR**	**TPR**	**TNR**	**FPR**	**FNR**
GBM	97.65	99.75	0.25	2.35	98.52	97.83	2.17	1.48	86.99	93.98	6.02	13.01	97.41	98.51	1.49	2.59
Random Forest	96.91	99.87	0.13	3.09	99.51	100.00	0	0.49	87.50	95.39	4.61	12.50	64.78	98.73	1.27	35.22
MLP	90.65	99.01	0.99	9.35	99.51	86.96	13.04	0.49	80.49	88.15	11.85	19.51	69.56	97.12	2.88	30.44
Naïve Bayes	82.66	97.94	2.06	17.34	96.06	100	0	3.94	46.71	87.67	12.33	53.29	69.41	92.45	7.55	30.59
SVM	75.18	99.89	0.11	24.82	98.52	100	0	1.48	85.00	81.07	18.93	15.00	55.82	96.43	3.57	44.18

**Table 7:** Performance comparison of AUC, F-score, and accuracy.

**Model**	**ISCX-URLs**			**PL-URLs**			**PhishGuard URLs**			**Suspicious-URLs**		
	**AUC**	**F1-score**	**Accuracy**	**AUC**	**F1-score**	**Accuracy**	**AUC**	**F1-score**	**Accuracy**	**AUC**	**F1-score**	**Accuracy**
GBM	99.10	98.92	99.46	98	99.01	98.39	93.20	91.56	93.87	98	97.93	97.96
Random Forest	98.89	98.65	99.33	100	99.75	99.60	94.10	92.47	94.25	82	78.02	81.75
MLP	95.87	94.11	97.52	93	98.30	97.19	88.15	84.39	86.74	83	80.66	83.47
Naïve Bayes	91.28	88.02	95.10	98	97.99	96.79	74.62	62.14	74.33	81	78.45	80.93
SVM	88.44	86.20	94.76	99	99.26	98.80	86.34	82.19	84.98	76	70.01	76.31

As shown in Table 6, which summarizes the TPR, TNR, FPR, and FNR, the GBM algorithm achieved the most balanced performance across all datasets. For instance, in the Suspicious-URLs dataset, GBM attained a TPR of 97.41% and a TNR of 98.51%, indicating high detection accuracy and minimal false predictions. Similarly, in the PL-URLs dataset, GBM achieved a TNR of 97.83% with a very low FPR of 2.17%, confirming its robustness and reliability in identifying phishing URLs.

The Random Forest model also delivered competitive results, achieving perfect TNR (100%) in the PL-URLs dataset and maintaining high accuracy across other datasets. However, its TPR of 64.78% on Suspicious-URLs suggests a trade-off between detection sensitivity and specificity in more complex environments. The MLP recorded high TPRs but exhibited slightly higher false positives, particularly an FPR of 13.04% in the PL-URLs dataset. In contrast, Naïve Bayes and SVM produced moderate results, performing adequately on simpler datasets such as ISCX and PL-URLs but showing reduced accuracy and higher FNR values on more challenging datasets like PhishGuard and Suspicious-URLs. Table 7 provides an overview based on AUC, F1-Score, and Accuracy. GBM consistently demonstrated superior performance, achieving an AUC of 98.70%, F1-Score of 98.38%, and Accuracy of 99.28% in the ISCX-URLs dataset, while sustaining high performance in others, including AUC = 90% and F1-Score = 89.07% on PhishGuard URLs. Random Forest also achieved remarkable results, particularly in the PL-URLs dataset with AUC = 100%, F1-Score = 99.75%, and Accuracy = 99.60%, demonstrating excellent precision. Although MLP achieved good performance overall, its lower AUC in complex datasets indicated sensitivity to data irregularities. Naïve Bayes and SVM performed consistently well in simpler datasets but exhibited performance degradation under complex or imbalanced data conditions.

The GBM algorithm emerged as the most effective and reliable model, consistently achieving high accuracy, precision, and balanced classification across all datasets. The integration of LIME for model interpretation further enhanced transparency by identifying critical features influencing model decisions, thereby improving explainability and trustworthiness. Random Forest ranked as a strong alternative with robust generalization, while MLP, Naïve Bayes, and SVM offered moderate yet dataset-dependent performance suitable for lightweight or resource-constrained scenarios.

**Figure 3:**
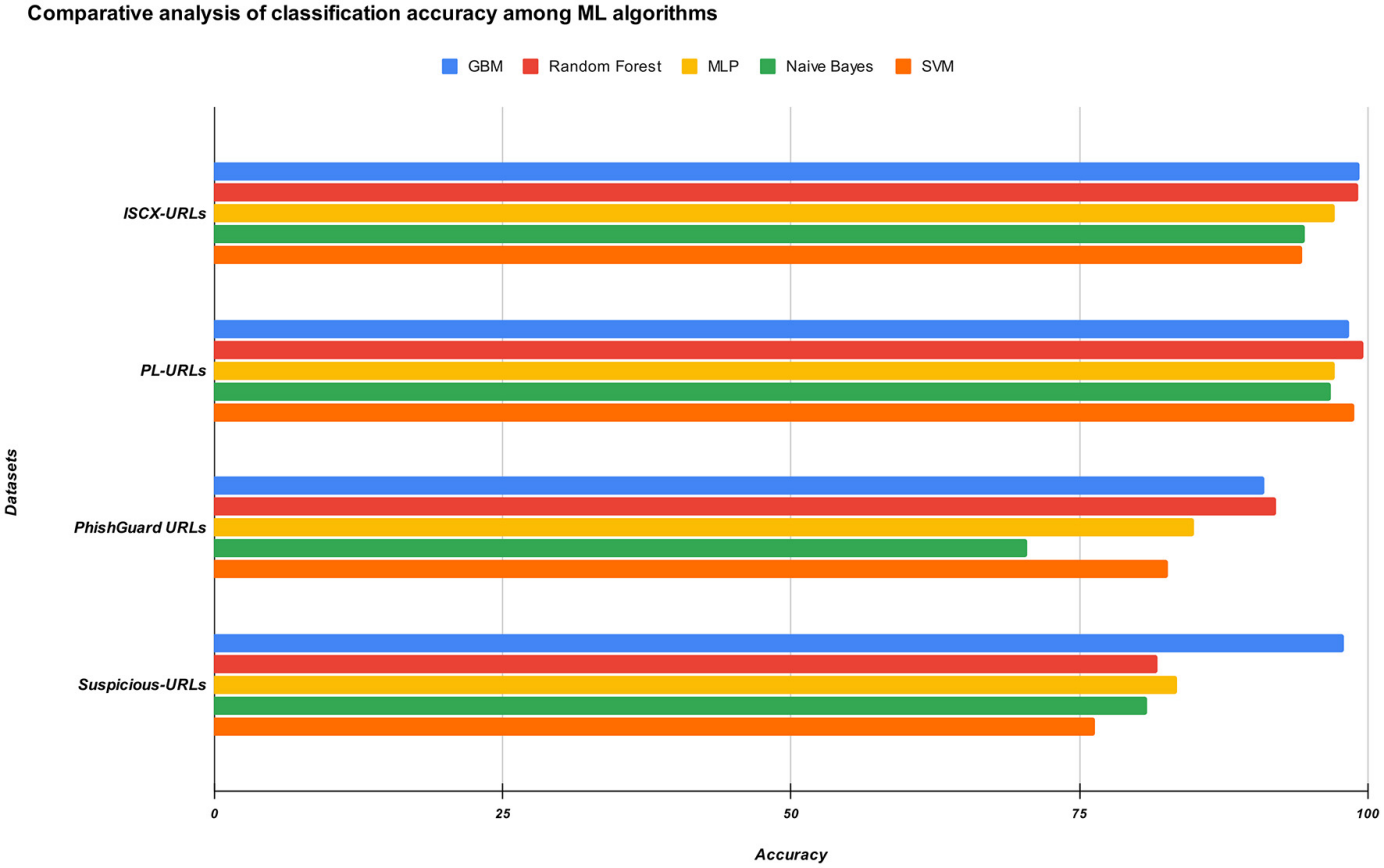
Comparative analysis of the experimental results of the proposed approach.

The comparative analysis in Figure 3 demonstrates that ensemble-based models, particularly GBM and Random Forest, deliver superior and more reliable accuracy across diverse phishing URL datasets. GBM consistently outperforms all other approaches, showing notable robustness on challenging datasets such as Suspicious-URLs, while Random Forest achieves peak performance on PL-URLs. In contrast, conventional classifiers such as SVM, MLP, and especially Naïve Bayes exhibit comparatively lower and less stable results, underscoring the effectiveness of ensemble learning methods in capturing complex patterns inherent in cyber-attack detection tasks.

#### 4.7.1 Interpretations using LIME and SHAP

This section presents an in-depth explanation of cyber-attack classification decisions using the LIME technique, applied to instances from various large-scale datasets. It provided feature-level interpretations that make the ML model's behavior transparent and interpretable for CyberSecurity professionals. Each instance is interpreted with respect to its explanation, domain insight, and importance, offering comprehensive insight into model reasoning and thought processes.

**Figure 4:**
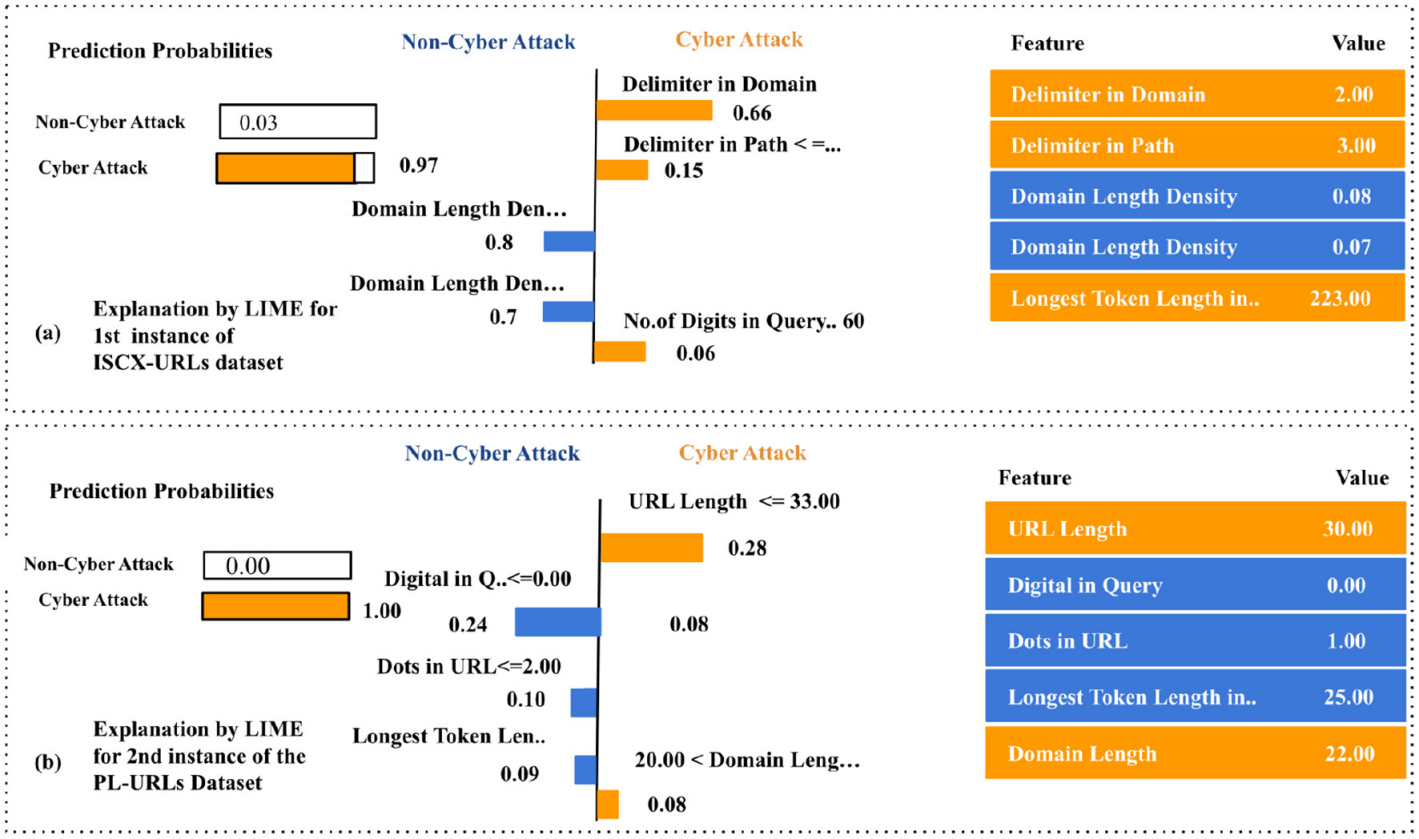
LIME local explanations for GBM predictions on **(a)** ISCX-URLs and **(b)** PL-URLs.

It should be noted from Figure 4a that the selected instance was classified as a cyber-attack with a predicted probability of 0.97. The LIME technique identified strong attack indicators, including the number of delimiters in the domain (2) and path (3), alongside a high longest token length of 223 characters. Two occurrences of low domain length density (0.08 and 0.07) contributed marginally toward a benign classification. Intuitively, URLs exhibiting structural complexity, such as an increased count of delimiters and extended tokens, are often associated with obfuscation strategies employed by attackers. A longest token length of 223 strongly implies manipulation to deceive users by mimicking legitimate domain patterns. On the contrary, features such as domain delimiters and token length were highly influential, with the domain delimiter alone contributing a weight of +0.66 toward the phishing class. In contrast, the benign-leaning DLD values, though present, were insufficient in magnitude to offset the phishing indicators, thereby justifying the classifier's decision.

Similarly, Figure 4b demonstrates that the instance was classified as a cyber-attack with full confidence [*P*(Class cyber-attack) = 1.00]. The LIME technique revealed that features such as URL length (30) and domain length (22) positively influenced the classification decision. On the other hand, non-cyber attack indicators included the absence of digits in the query, a low dot count in the URL (1), and a moderate longest token length (25). Although fewer dots and the absence of numeric characters typically correlate with legitimate URLs, a domain length greater than 20, coupled with a moderate URL length, suggests potential risk. The URL length feature provided a significant weight of +0.28 toward the cyber-attack class. Despite benign contributions from structural simplicity, the collective weight of attack indicators surpassed the benign cues, leading to a confident cyber-attack classification.

**Figure 5:**
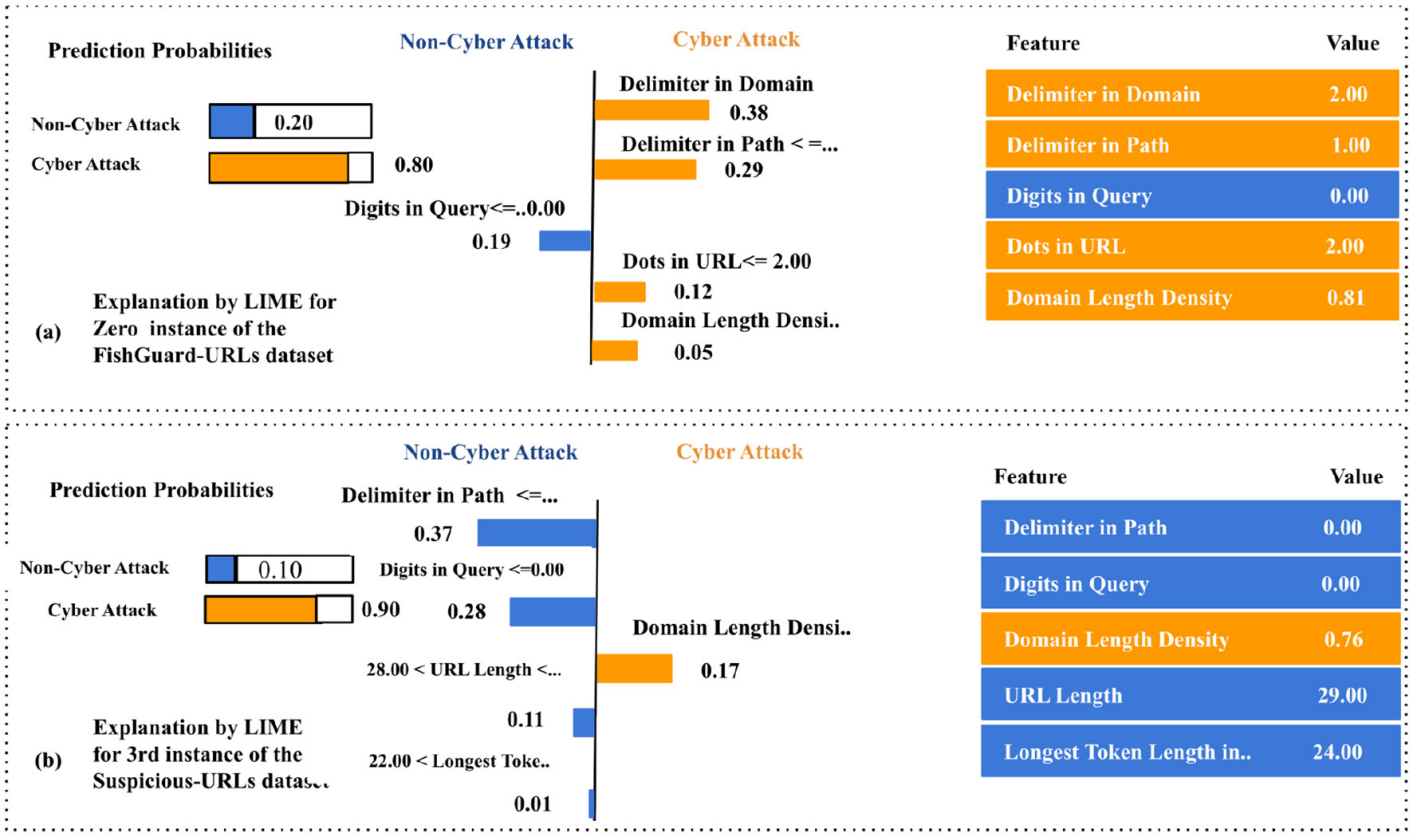
LIME local explanations for GBM predictions on **(a)** PhishGuard and **(b)** suspicious-URLs.

It should be noted from Figure 5a that the instance was classified as a cyber-attack with a confidence score of 0.80. The LIME technique attributed this decision to features such as delimiters in the domain (2, +0.38), delimiters in the path (1, +0.29), and dots in the URL (2, +0.12). A higher domain length density (0.81, +0.05) further contributed to this class. A single benign feature—absence of digits in the query (0)—had a negative weight of −0.19 toward the non-cyber attack class. In general, malicious URLs often integrate numerous delimiters and high-density structures to emulate legitimate domains while embedding malicious elements. A domain length density close to 1 indicates that the domain portion of the URL is abnormally lengthy compared to the full URL, signaling obfuscation. Although this signal contributed slightly, the attack-oriented structural patterns held dominant influence. The balance of feature weights highlights the classifier's sensitivity to hierarchical and syntactic URL components in assessing legitimacy.

**Figure 6:**
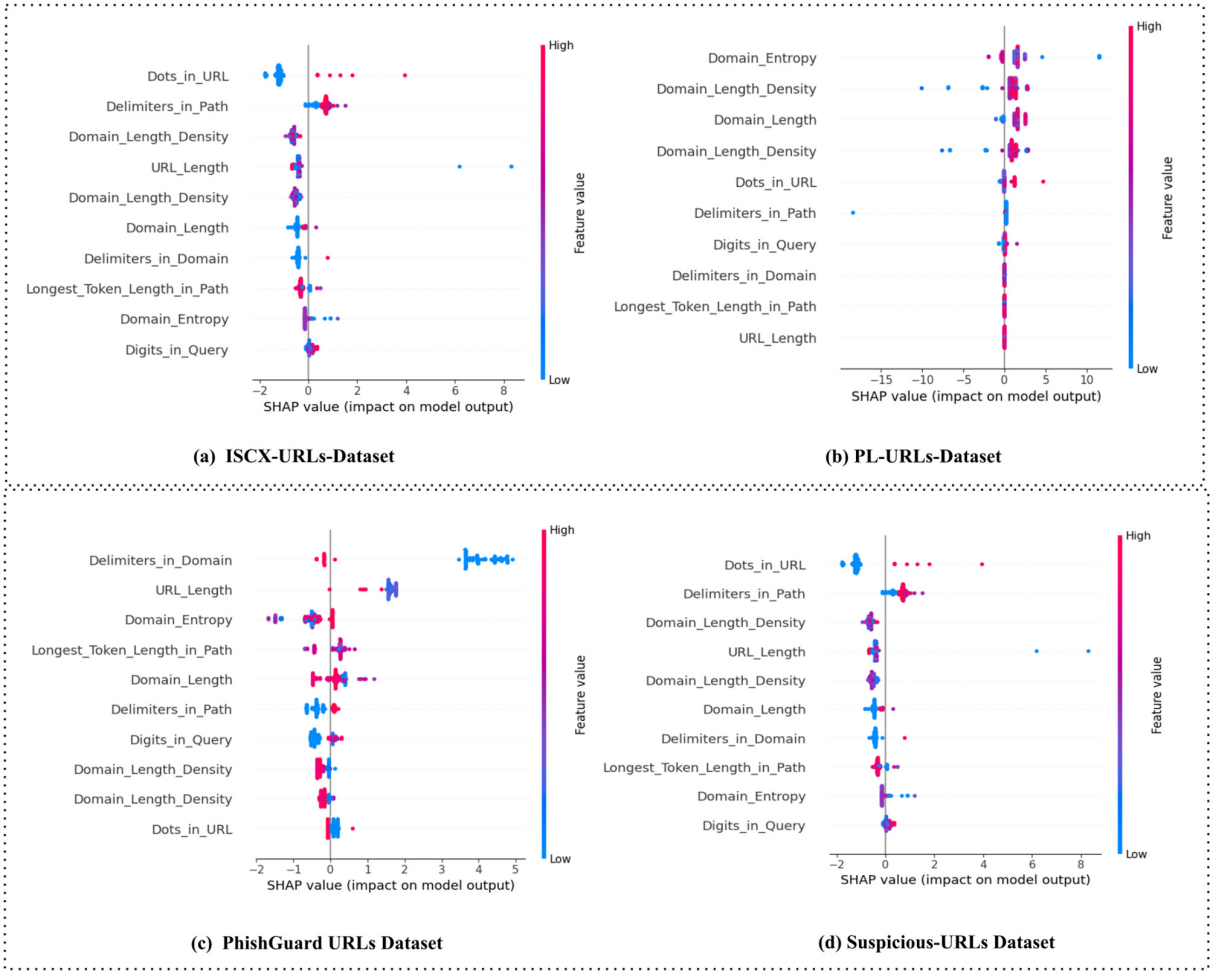
SHAP-driven explanations for dataset predictions. **(a)** ISCX-URLs-dataset. **(b)** PL-URLs-dataset. **(c)** PhishGuard URLs dataset. **(d)** Suspicious-URLs dataset.

Similarly, Figure 5b demonstrates that the selected instance from the Suspicious-URLs dataset was labeled cyber-attack with a probability of 1.00. Influential features included the number of delimiters in the path (2, +0.53), domain entropy (2.52, +0.19), and an anomalous top-level domain (0.00, +0.13). Minor benign signals were observed in domain length density (0.08, −0.28) and longest token length (61, −0.07). In general, entropy-based patterns are especially effective in capturing randomness within domain structures. Malicious URLs often maximize entropy to bypass domain-based filtering systems. Additionally, non-standard or null TLDs are recognized as indicators of suspicious domain registration practices. Hence, the path delimiter and entropy scores were the most influential indicators of cyber-attack. Despite the presence of benign-like patterns in domain structure, their relative weights were insufficient to counteract the strength of high-entropy signals and structural anomalies. On the contrary, Figure 6 visualizes SHAP summary plots for four cyber-attack detection datasets: ISCX-URLs, PL-URLs, PhishGuard URLs, and Suspicious-URLs. In each plot, features are ranked vertically by their mean absolute SHAP value, indicating their relative influence on the model's predictions. The horizontal axis represents SHAP values, where positive values shift the prediction toward the cyber-attack class, and negative values favor the benign class. Feature values are color-coded, with red denoting higher values and blue indicating lower values.

It is evident from Figure 6a that the number of dots in the URL and the number of delimiters in the path emerge as the strongest attack indicators. Low Domain Length Density also contributes toward attack predictions, while higher DLD values lean toward benign classification. URL length and the longest token length in the path exert a moderate positive influence on attack likelihood. On the other hand, Figure 6b visualizes that domain entropy and low DLD are the most influential features. Longer domain lengths and an increased number of path delimiters also push predictions toward attack. Benign tendencies are associated with low dot counts and fewer delimiters in the domain. Similarly, Figure 6c illustrates that the number of domain delimiters and long URLs dominate cyber-attack classification. Elevated domain entropy and long path tokens further reinforce attack predictions, whereas higher DLD values and fewer dots in the URL provide a weaker, benign influence. In addition, Figure 6d demonstrates that dot count in the URL and path delimiters are the most impactful cyber-attack predictors. URL length, low DLD, and long path tokens contribute additional phishing evidence, while benign-leaning signals such as higher DLD and lower domain entropy remain relatively minor.

The LIME technique is a *post-hoc* explainability approach that interprets individual predictions made by ML models, as outlined in Algorithm 8. Rather than explaining the model globally, LIME focuses on generating local surrogate models that approximate the decision boundary near a specific instance. For each selected URL sample, a set of 5,000 perturbed observations was generated around the instance using Gaussian sampling. These samples were evaluated by the trained classifier, and a TabularExplainer was used to train a local linear surrogate model with a kernel width of 0.75 and auto feature selection. The process was repeated three times using a fixed random seed (42) to ensure reproducibility. This configuration allowed consistent local interpretations across all models, providing insight into which URL-based features most influenced classification decisions.

Algorithm 8 LIME explanation for URL classification. 
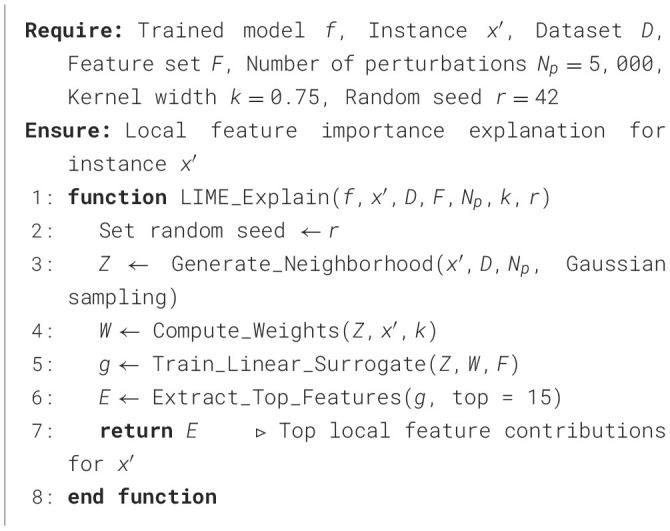

On the other hand, the SHAP framework provides a complementary, theoretically grounded explainability method based on cooperative game theory, as described in Algorithm 9. SHAP computes the contribution of each feature to a model's prediction by treating each feature as a player in a cooperative game, where the overall prediction is the payout. In this study, TreeExplainer was applied for tree-based models, while KernelExplainer was used for non-tree models. A background dataset of 100 randomly selected training instances was used to estimate expected values.

Algorithm 9 SHAP explanation for URL classification. 
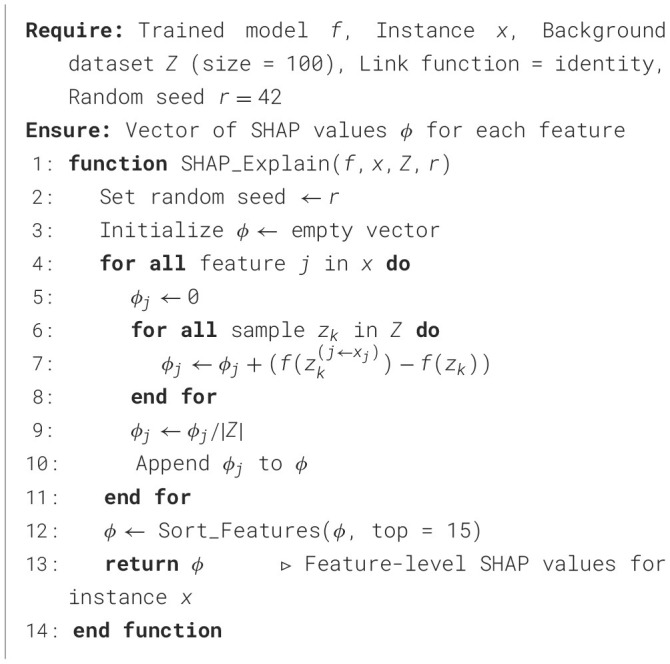

Both LIME and SHAP were executed under identical pre-processing and feature-scaling conditions across all datasets. The combination of LIME's local linear approximations and SHAP's globally consistent feature attribution provides both micro- and macro-level interpretability for phishing-detection models, enhancing the transparency and accountability of cyber-threat intelligence systems.

#### 4.7.2 Decision-making of LIME and SHAP

The LIME and SHAP techniques are complementary techniques for understanding how ML models make predictions. In the context of cyber-attack detection, both methods enhance interpretability by identifying the features that most significantly influence classification decisions. LIME provides instance-level explanations, highlighting how specific features affect the prediction for an individual URL, while SHAP offers a unified, game-theoretic approach to quantify feature contributions across both local and global contexts. Together, they transform opaque “black box” models into transparent systems, enabling CyberSecurity analysts to interpret and validate results more effectively.

In this study, LIME and SHAP were applied to predictions generated by the ML classifiers across multiple URL datasets. Both methods consistently identified features such as domain length, number of delimiters in the domain, and domain entropy as critical for accurate classification. For malicious URLs, these features generally exhibited strong positive contributions, while for legitimate URLs, they showed a negative influence. SHAP further reinforced these findings by providing consistent feature attributions across the entire dataset, confirming the robustness and reliability of these key indicators.

The combined use of LIME and SHAP significantly improved model interpretability and increased trust in predictions. LIME's real-time, instance-level insights help analysts investigate individual decisions, while SHAP's global perspective ensures consistency and stability in feature importance rankings. This dual-layer interpretability is particularly valuable in CyberSecurity, where decision-making must be both precise and explainable. By integrating these methods, analysts can detect potential misclassifications, understand model behavior at multiple levels, and deploy cyber-attack detection systems confidently in high-stakes operational environments.

## 5 Discussion

Despite the little use of ML for cyber-attack detections in literature, its application to analyzing URL patterns has proven highly effective (Karim et al., 2023). ML equips CyberSecurity professionals with enhanced decision-making capabilities through accurate predictions, classifications, and visualizations of critical URL features. However, many high-performance ML algorithms operate as black boxes, making it difficult to interpret their internal decision-making processes. This lack of transparency presents a significant challenge, particularly in safety-critical domains like CyberSecurity, where trust and explainability are essential.

To address the cyber-attack detection challenge in the Big Data environment, we applied five black box ML algorithms, including GBM, RF, MLP, NB, and SVM, accompanied by XAI techniques to generate interpretable insights into the cyber-attack detection process. The findings confirm that LIME and SHAP are valuable tools for visualizing and understanding black box ML models' behavior in URL classification, achieving precision levels exceeding 99% in cyber-attack URL detections.

More recently, Karim et al. (2023) proposed an ML-based phishing detection approach using URLs, introducing a hybrid model combining Logistic Regression, Support Vector Machine, and Decision Tree (LR+SVC+DT) through both soft and hard voting mechanisms, achieving approximately 95% accuracy. In contrast, our approach achieved accuracy levels up to 99%, while incorporating LIME to provide interpretable, instance-level explanations of model decisions. Similarly, Imtiaz et al. (2025) enhanced classifier performance through genetic algorithms and particle swarm optimization, reporting that a Multinomial Naïve Bayes model paired with a genetic algorithm yielded the best results. Our findings also confirm the effectiveness of Naïve Bayes, but we further enhanced model transparency via LIME and benchmarked it against other widely used ML algorithms.

In another study, Jouini et al. (2024) extracted key features from phishing websites across seven email datasets, where Random Forest Max Vote Classifier and Decision Tree models achieved 97.73% accuracy. Likewise, our research identified Nine essential features and achieved an overall accuracy of 98%, with GBM delivering the highest performance. In addition, emphasized URL structure, domain age, SSL certificate, page length, and JavaScript presence as key indicators for cyber-attack detection, finding Random Forest most effective. Similarly, proposed a Random Forest-based approach was proposed that achieved an F1 score of 97%, accuracy of 97%, and recall of 99%, with notable efficiency and low computational cost. In line with these studies, our work used Nine critical features and demonstrated that GBM consistently outperformed other models.

This research underscores the strong potential of ML in cyber threat detection, particularly in Big Data environments where scalability and real-time analysis are crucial. Leveraging URL pattern analysis in such frameworks enables efficient identification of cyber threats, while integrating LIME significantly improves decision transparency by providing clear, instance-level insights into classification outcomes. Comparative analysis across prior studies confirms that features such as Domain Length and Number of Delimiters in Domain consistently play a decisive role in distinguishing between malicious and legitimate URLs.

ML and XAI are transformative in CyberSecurity, enhancing the ability to detect, interpret, and mitigate sophisticated threats. The models used in this study—including GBM, RF, SVM, MLP, and NB are well-suited for processing large, heterogeneous, and high-velocity datasets typical of CyberSecurity applications. While these models automate detection and improve classification accuracy, the black box nature of many ML models limits their applicability in high-stakes environments where accountability and explainability are critical. XAI addresses this limitation by making model predictions more transparent and interpretable. By clarifying the reasoning behind predictions, XAI enables CyberSecurity analysts to validate outputs, improve trust in automated systems, and ensure compliance with regulatory and ethical standards. The ability to trace and justify automated decisions strengthens the responsible adoption of AI in security-critical domains.

Our findings are based on four publicly available datasets, which may constrain generalizability. Future research should incorporate more diverse and multilingual datasets to improve adaptability against evolving phishing tactics. Developing real-time cyber-attack detection systems that sustain high accuracy with minimal latency is vital for resource-constrained environments such as mobile devices and IoT platforms. Enhancing explainability remains a priority, requiring XAI tools capable of delivering real-time, intuitive explanations that analysts can interpret effectively. Extending cyber-attack detection to social media and messaging platforms, while ensuring robustness against adversarial manipulation, will be essential for maintaining the trustworthiness and resilience of ML-driven CyberSecurity systems.

## 6 Conclusion

The rapid expansion of internet usage and the evolution of digital technologies have simultaneously created new opportunities and intensified CyberSecurity risks. Among these, phishing remains a pervasive and adaptive threat, exploiting unsuspecting users through deceptive domains and malicious links that target sensitive information. As the volume and sophistication of fraudulent websites increase, distinguishing legitimate from malicious domains has become a formidable challenge requiring scalable, accurate, and transparent detection mechanisms.

This study addressed these challenges by integrating black box ML models with XAI techniques in a Big Data-driven CyberSecurity framework. Experimental findings revealed that GBM consistently outperformed RF, MLP, NB, and SVM across multiple URL datasets. To mitigate the interpretability limitations of black box models, two complementary XAI approaches, LIME and SHAP, were applied, providing both local and global explanations of GBM predictions. These methods consistently highlighted domain length, number of delimiters, and domain entropy as the most influential features in cyber-attack detection, thereby uncovering structural patterns of malicious URLs. Such interpretability not only enhances trust in automated systems but also supports expert validation and informed decision-making in operational CyberSecurity contexts.

In conclusion, the integration of high-performing black box ML algorithms with robust explainability techniques significantly improves both the accuracy and transparency of cyber-attack detection systems. This dual emphasis ensures that detection models remain not only effective in identifying threats but also trustworthy, interpretable, and compliant with operational and regulatory requirements. Future research should extend validation to larger and more heterogeneous datasets, while also exploring real-time detection frameworks optimized for resource-constrained environments, including mobile and IoT platforms. Expanding detection capabilities to encompass social media, messaging services, and emerging communication channels will further enhance the robustness and adaptability of cyber defense mechanisms.

## Data Availability

The original contributions presented in the study are included in the article/supplementary material, further inquiries can be directed to the corresponding author.

## Footnotes

1
https://drive.google.com/drive/u/0/folders/1CC9h6fpslNGeZlWEVOlwjTQ9bQ-DBXzV
